# Increased Frequency of Pre–Pro B Cells in the Bone Marrow of New Zealand Black (NZB) Mice: Implications for aDevelopmental Block in B Cell Differentiation

**DOI:** 10.1080/1044667021000003961

**Published:** 2002-03

**Authors:** Zhe-Xiong Lian, Hiroto Kita, Tomoyuki Okada, Tom Hsu, Leonard D. Shultz, Kenneth Dorshkind, Aftab A. Ansari, Susumu Ikehara, Mitsuru Naiki, M. Eric Gershwin

**Affiliations:** 1 Division of Rheumatology Allergy and Clinical Immunology University of California at Davis School of Medicine TB 192, One Shields Avenue Davis CA 95616 USA; 2 Institute of Bio-Active Science Nippon Zoki Pharmaceutical Co., Ltd. Kinashi, Yashiro-Cho Kato-gun Hyogo 673-1461 Japan; 3 The Jackson Laboratory 600 Main Street Bar Harbor ME 04609 USA; 4 Department of Pathology and Laboratory Medicine and Josson Comprehensive Cancer Center School of Medicine University of California Los Angeles CA 90095 USA; 5 Department of Pathology Emory University School of Medicine Atlanta GA 30322 USA; 6 fFirst Department of Pathology Transplantation Center Kansai Medical University Moriguchi Osaka Japan

## Abstract

Reductions in populations of both Pre-B cell (Hardy fractions D) and Pro-B cells (Hardy fractions B–C) have been described in association with murine lupus. Recent studies of B cell populations, based on evaluation of B cell differentiation markers, now allow the enumeration and enrichment of other stage specific precursor cells. In this study we report detailed analysis of the ontogeny of B cell lineage subsets in New Zealand black (NZB) and control strains of mice. Our data suggest that B cell development in NZB mice is partially arrested at the fraction A Pre–Pro B cell stage. This arrest at the Pre-Pro B cell stage is secondary to prolonged lifespan and greater resistance to spontaneous apoptosis. In addition, expression of the gene encoding the critical B cell development transcription factor BSAP is reduced in the Pre–Pro B cell stage in NZB mice. This impairment may influence subsequent B cell development to later stages, and thereby accounts for the down-regulation of the B cell receptor component *Igα* (mb-1). Furthermore, levels of expression of the *Rug2, λ5* and *Igβ* (B29) genes are also reduced in Pre–Pro B cells of NZB mice. The decreased frequency of precursor B cells in the Pre–Pro B cell population occurs at the most primitive stage of B cell differentiation.


*Developmental Immunology*, 2002 Vol. 9 (1), pp. 35–45

Taylor & Francis healthsciences

### Increased Frequency of Pre–Pro B Cells in the Bone Marrow of New Zealand Black (NZB) Mice: Implications for a Developmental Block in B Cell Differentiation

ZHE-XIONG LIAN[a], HIROTO KITA[a], TOMOYUKI OKADA[a,b], TOM HSU[a], LEONARD D. SHULTZ[c], KENNETH DORSHKIND[d], AFTAB A. ANSARI[e], SUSUMU IKEHARA[f], MITSURU NAIKI[b] and M. ERIC GERSHWIN[a,*]

[a]*Division of Rheumatology, Allergy and Clinical Immunology, University of California at Davis School of Medicine, TB 192, One Shields Avenue, Davis, CA 95616, USA;* [b]*Institute of Bio-Active Science, Nippon Zoki Pharmaceutical Co., Ltd., Kinashi, Yashiro-Cho, Kato-gun, Hyogo 673-1461, Japan;* [c]*The Jackson Laboratory, 600 Main Street, Bar Harbor, ME 04609, USA;* [d]*Department of Pathology and Laboratory Medicine and Josson Comprehensive Cancer Center, School of Medicine, University of California, Los Angeles, CA 90095, USA;* [e]*Department of Pathology, Emory University School of Medicine, Atlanta, GA 30322, USA;* [f]*First Department of Pathology, Transplantation Center, Kansai Medical University, Moriguchi, Osaka, Japan*

Reductions in populations of both Pre-B cell (Hardy fractions D) and Pro-B cells (Hardy fractions B–C) have been described in association with murine lupus. Recent studies of B cell populations, based on evaluation of B cell differentiation markers, now allow the enumeration and enrichment of other stage specific precursor cells. In this study we report detailed analysis of the ontogeny of B cell lineage subsets in New Zealand black (NZB) and control strains of mice. Our data suggest that B cell development in NZB mice is partially arrested at the fraction A Pre–Pro B cell stage. This arrest at the Pre–Pro B cell stage is secondary to prolonged lifespan and greater resistance to spontaneous apoptosis. In addition, expression of the gene encoding the critical B cell development transcription factor BSAP is reduced in the Pre–Pro B cell stage in NZB mice. This impairment may influence subsequent B cell development to later stages, and thereby accounts for the down-regulation of the B cell receptor component *Igα* (mb-1). Furthermore, levels of expression of the *Rug2*, *λ5* and *Igβ* (B29) genes are also reduced in Pre–Pro B cells of NZB mice. The decreased frequency of precursor B cells in the Pre–Pro B cell population occurs at the most primitive stage of B cell differentiation.

*Keywords*: Autoimmunity; B Lymphocytes; Lupus; New Zealand black mice

### INTRODUCTION

Rapid progress is being made in defining lineage specific precursor cells that are intermediates in the pathway of B cell differentiation (Payne *et al.*, 1999; Akashi *et al.*, 2000). Development of mature B cells from multipotential stem cells is accompanied by qualitative and/or quantitative differences in the expression of cell surface molecules. Such differences permit enumeration, depletion and enrichment of stage-specific precursor cells. Merchant *et al.*(1995; 1996) fractionated bone marrow cells (BMC) from lupus-prone mice according to stage specific fractions (Hardy and Hayakawa, 1991) and demonstrated an age-dependent reduction of both Pre- (Fr. D) and Pro-B cells (Fr. B–C) in the bone marrow (BM). Herein, we performed a detailed study of the ontogeny of each sub-stage specific lineage of B cells from New Zealand black (NZB) mice. Our data reveal an accumulation, rather than a reduction, of the most immature B lineage cells, referred to as Pre–Pro B cells. Furthermore, the increased frequency of Pre–Pro B cells was secondary to a decreased rate of apoptosis. Thus, the decreased frequency of precursor B cells in NZB mice occurs at the most primitive stage of B cell differentiation may be secondary to abnormalities in the gene(s) that control B cell lineage differentiation.

### MATERIALS AND METHODS

### Mice and Cell Preparation

Female NZB/BlnJ, BALB/CJ, C3H/HeJ, C57BL/6J mice, aged 1–8 months, were obtained from Jackson Laboratory (Bar Harbor, ME) and subsequently maintained by the Animal Resource Service of the University of California at Davis. BMC were obtained by flushing two femurs and tibiae with PBS containing 0.2% bovine serum albumin

*Corresponding author. Tel.: +1-530-752-2884. Fax: +1-530-752-4669. E-mail: megershwin@ucdavis.edu

ISSN 1044-6672 print © 2002 Taylor & Francis Ltd
DOI: 10.1080/1044667021000003961

(BSA) utilizing a 25-gauge needle. Single cell suspensions were washed and viable cells quantitated and confirmed using trypan blue exclusion. The data presented in all experiments was replicated in three separate experiments using 3–4 mice per group, unless otherwise noted.

### Antibodies

FITC-, PE- or biotin-conjugated mAb RA3.6B2 (anti-CD45R, B220), S7 (anti-CD43), GK1.4 (anti-CD4), 53-6.7 (anti-CD8), M1/70 (anti-CD11b, Mac-1), RB6-8C5 (anti-Ly6G, Gr-1), TER-119 (anti-TER-119), E13-161.7 (anti-Ly6A/E, Sca-1), 2B8 (anti-CD117, c-Kit), 1D3 (anti-CD19), 53-7.3 (anti-CD5), 17A2 (anti-CD3), 2.4G2 (anti-CD32/CD16 (Fc$\gamma$II/IIIR)), PK136 (anti-NK1.1) were obtained from BD PharMingen (San Diego, CA). J11d (anti-CD24, (heat stable antigen) HSA), CT-CD8$\alpha$ (anti-CD8), CT-CD4 (anti-CD4) and Streptavidin TC® were purchased from Caltag laboratories (Burlingame, CA).

### Immunofluorescence Labeling and FACS Analysis

Immunofluorescence labeling was performed as described by Lian *et al.*(1997). Expression of cell surface antigens was measured by three-color flow cytometry analysis. Briefly, BMC were aliquoted ($10^6$) into tubes and preincubated with CD32/CD16 (Fc Block™) at 4°C for 5 min. FITC-labeled anti-CD43, CD4 or IgM and PE-labeled anti-B220, CD19, CD5, HSA or c-kit together with biotin-labeled anti-CD3, TcR-$\alpha\beta$, Thy1.2, Mac-1, Gr-1, NK1.1, Sca-1, CD4, CD8 or B220 were added directly to cells in Fc Block™ at 4°C for 30 min. Cells were then washed and subsequently incubated with streptavidin TRI®. The frequency of cells expressing individual and/or sets of cell surface markers and the mean density of expression of such markers was determined by analysis of a minimum of 50,000 cells utilizing a FACScan flow cytometer (Becton Dickinson) and Cell Quest software.

NZB bone marrow B lineage cells can be divided into distinct maturational stages based on surface staining for CD43 and sIgM or HSA as reported by Hardy and Hayakawa (1991). The maturational subsets are identified alphabetically and represent increasing stages of differentiation from the Pre–Pro B cell subset stage (Fr. A: $B220^+CD43^+HSA^-CD19^-$) → Pro-B cell subset stage (Fr. B–C: $B220^+CD43^+HSA^+CD19^+$) → Pre-B cell subset stage (Fr. D: $B220^+CD43^-sIgM^-CD19^+$) and immature B cell subset stage (Fr. E–F: $B220^+CD43^-sIgM^+CD19^+$) as noted in Fig. 1. We specifically quantitated $NK1.1^+$ cells in the Pre–Pro B cell population of NZB, BALB/c and C57BL/6 mice, based on earlier work suggesting that $NK1.1^+$ cells may be a minor population in the Pre–Pro B population (Rolink *et al.*, 1996).

### Depletion of Immature/Pre/Pro (Fr. B–F) B Cells

BMC were collected and layered onto a NycoPrep™ (NycoMed Pharma As, Oslo, Norway) discontinuous density gradient. After centrifugation at 800*g* for 25 min, cells with a density of $1.066 < p < 1.077$ were collected (Lian *et al.*, 1999). The low-density cells were treated with a mixture of rat mAbs against mouse sIgM, CD24 (HSA) and CD19 followed by incubation with anti-rat IgG-conjugated magnetic-beads (Dynabeads®). Passage through a magnetic field was utilized to deplete the Immature/Pre/Pro B (Fr. B–F) cells.

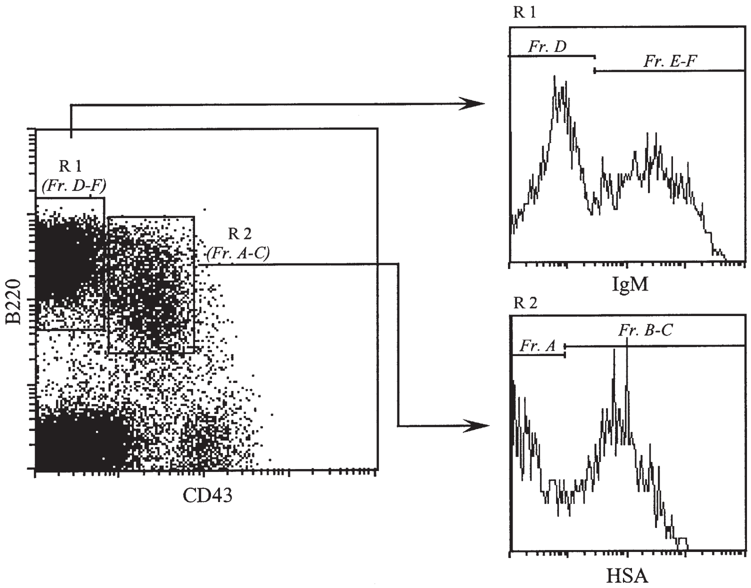

FIGURE 1 Flow cytometric analysis of bone marrow showing identification of B lineage subsets in NZB mice. CD45R $(B220)^+CD43^+$ (R2: Fr. A–C) were resolved into the Pro-B cell subset (Fr. B–C) and the Pre–Pro B cell subset (Fr. A) based on expression of HSA. CD45R $(B220)^+CD43^-$ cells (R1: Fr. D–F) were resolved into Immature B cells (Fr. E–F) and Pre-B cell subsets (Fr. D) based on the expression of IgM.

### Cell Cycle Analysis

Cell cycling was detected by the BrdU Flow Kit (BD PharMingen, San Diego, CA). Mice were injected i.p. with 1 mg BrdU dissolved in PBS and were thereafter fed drinking water containing 1 mg/ml BrdU for different periods of time. The drinking water was light protected and replaced with fresh BrdU-containing water every 2 days. Briefly, purified $IgM^-$/$HSA^-$/CD $19^-$ BMC were stained with PE-anti B220 mAb, fixed with Cytofix/Cytoperm Buffer, and permeabilized with Cytoperm Plus Buffer. Cells were then incubated again with Cytofix/Cytoperm Buffer, followed by treatment with DNase to expose the BrdU epitopes. Finally, immunofluorescent staining was performed with FITC conjugated anti-BrdU (for defining the frequency of dividing cells) and 7-AAD (for measurement of total DNA content), and analyzed by FACScan.

### Detection of Apoptotic Cells

The frequency of Pre–Pro B (Fr. A) cells undergoing apoptosis was detected by Annexin V staining (BD PharMingen, San Diego, CA). After depletion of Immature/Pre/Pro (Fr. B–F) B cells, $5 \times 10^5$ $IgM^-$/ $HSA^-$/$CD19^-$ fresh or cultured cells were resuspended in binding buffer (10 nM HEPES/NaOH, pH 7.4, 140 mM NaCl, and 2.5 nM $CaCl_2$). Thence, the cells were incubated with PE-conjugated Annexin V for 15 min at room temperature in the dark, washed and analyzed using the FACScan.

### RNA Isolation and Reverse Transcription PCR

Total RNA for cDNA synthesis was prepared from freshly enriched Pre–Pro B (Fr. A) cells as described above. Briefly, the low-density Immature/Pre/Pro (Fr. B–F) B cells ($sIgM^+$, $HSA^+$, $CD19^+$) were first depleted using Dyna magnetic beads. Such enriched $Lin^-$ cells were subsequently incubated with CD45R (B220) microbeads and subjected to further enrichment using a cell sorter (Miltenyi Biotec Inc., Auburn, CA). The resulting $B220^+$ Pre–Pro B cells had a purity greater than 95% (Fig. 2). RNA was extracted utilizing the RNAeasy Mini kit (QIAGEN Inc., Santa Clarita, CA) and eluted into DEPC-treated $H_2O$ and stored at −70°C. This RNA was used to synthesize first strand cDNA using Superscript II reverse transcriptase (RT; GIBCO life Technologies, Gaithersburg, MD), 1 mM dNTPs, 1 μg random hexameric oligonucleotides, and the supplied RT buffer (GIBCO BRL). PCR assays were carried out using the following primer pairs: *β-actin*, 5′-CCT AAG GCC AAC CGT GAA AAG, 5′-TCT TCA TGG TGC TAG GAG CCA; $\mu_0$, 5′-AAC ATC TGA GTT TCT GAG GCT TGG, 5′-TCA TCT GAA CCT TCA AGG ATG CTC; *E2A*, 5′-CAT CCA TGT CCT GCG AAG CCA, 5′-TTC TTG TCC TCT TCG GCG TC; *Id*, 5′-TCC AAC TTC TTG TTC TCT TCC C, 5′-CAC AAG ATG CGA TCG TCG; BSAP, 5′-TCC TCG GAC CAT CAG GAC AG, 5′-CCT GTT GAT GGA GCT GAC GC; *λ5*, 5′-CTT GAG GGT CAA TGA AGC TCA GAG TA, 5′-CTT GGG CTG ACC TAG GAT TG; *mb-1*, 5′-GCC AGG GGG TCT AGA AGC,

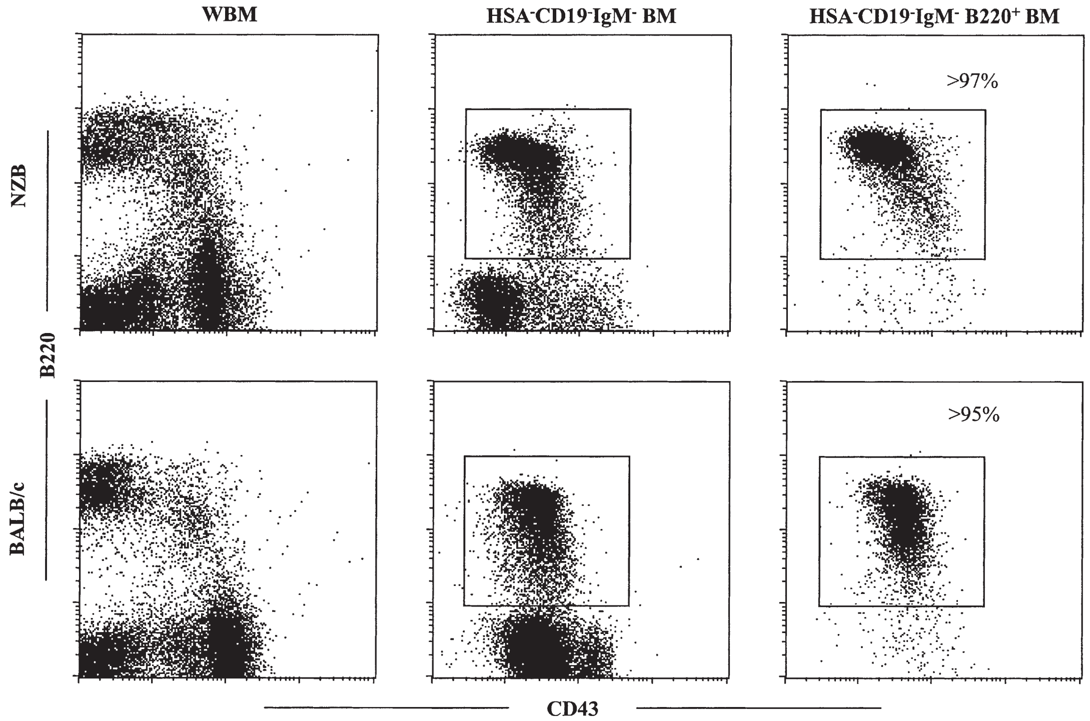

FIGURE 2 Whole BMC from NZB mice were enriched after discontinuous density gradent centrifugation; the low-density $IgM^+$, $HSA^+$, $CD19^+$ cells were first depleted using Dyna magnetic beads. Such enriched $Lin^-$ cells were subsequently incubated with CD45R (B220) microbeads and subjected to further enrichment using a cell sorter. The resulting $B220^+$ Pre–Pro B cells had a purity greater than 95%.

5′-TCA CTT GGC ACC CAG TAC AA; *B29*,5′-TAA GTC TAG AAG TTC CGT GCC ACA GCT GTC, 5′-CAC TGA ATT CCC AAG GAA GCC CTT GTT CCC; *Bcl-X_l*, 5′-TGA TTC CCA TGG CAG CAG TGA, 5′-AAC CAC ACC AGC CAC AGT CAT-3′.

### Statistical Analysis

Values were determined to be statistically significant by ANOVA test or by unpaired Student's *t*-test.

### RESULTS

### Bone Marrow B Cell Subset Changes

In order to determine the frequency and absolute number of cells at various stages of B cell development in NZB mice of different ages, bone marrow samples from 1, 2, 4 and 8 month old mice were examined. As shown in Fig. 3, the frequency and absolute numbers of cells in fractions B–F decreased with age. By 8 months of age, the proportion (and number) of Immature B cells (Fr. E–F) decreased from 6.1 to 2.2% ($3.0 \times 10^6$ to $1.1 \times 10^6$), Pre-B cells (Fr. D) from 11.6 to 3.2% ($5.8 \times 10^6$ to $1.5 \times 10^6$) and Pro-B cells (Fr. B–C) decreased from 4.3 to 0.2% ($2.1 \times 10^6$ to $0.5 \times 10^6$). In contrast, there was a marked increase in the frequency (from 3.6 to 6.7%) and absolute numbers ($1.8 \times 10^6$ to $2.7 \times 10^6$) of Pre–Pro B cells (Fr. A) from 1 to 2 months of age. Pre–Pro B cells at 8 months of age, however, showed an age associated decline, but still remained higher than levels at 1 month of age.

As shown in Fig. 4A, a similar analysis of B cell differentiation in BALB/c, C3H, and C57BL/6 mice demonstrated that the trend was unique to NZB mice. The data in Fig. 4 also show that the decline in the frequency and absolute number of immature and Pre-B cells in bone marrow of NZB mice is greater than that seen in normal mice. Unexpectedly, the Pre–Pro B cell populations are unusually expanded in NZB mice, and significantly higher at 8 months in comparison with control mice (Fig. 4D). It has been previously reported that NK1.1$^+$ cells may be included in a minor population of a Pre–Pro B cell population (Rolink *et al.*, 1996). In preliminary experiments, we detected a similar frequency of NK1.1$^+$ Pre–Pro B cells in age-matched NZB mice and C57BL/6 mice. However, NK1.1 are not expressed in BALB/c mice (data not shown). Further, the NK1.1 population was depleted before our analysis.

### BrdU Labeling of Pre–Pro B Cells

It was reasoned that the increased number of Pre–Pro B cells in the bone marrow of NZB mice could be secondary to an increased level of proliferation of cells at this stage of B cell maturation. In order to examine this possibility, we analyzed the percentages of BrdU$^+$ Pre–Pro B cells in bone marrow from NZB, and for comparison, BALB/C and C57BL/6 mice. Attempts were made in initial experiments to assess the frequency of BrdU$^+$ Pre–Pro B cells in unfractionated bone marrow, but the low numbers of Pre–Pro B cells made this analysis difficult. Therefore, after low-density cell isolation, the IgM$^+$/HSA$^+$/CD19$^+$ BMC were depleted using magnetic beads as described in the "Materials and Methods" section, prior to analysis (Fig. 5). As shown in Fig. 6 and Table I, the frequency of BrdU$^+$ cells in NZB Pre–Pro B cells is lower than in normal mice. Two hours after the injection of BrdU, only 0.9% of the Pre–Pro B cells were BrdU$^+$ in the NZB mice, while almost 2% of the Pre–Pro B cells from BALB/c mice were BrdU$^+$. In addition, while 90% of Pre–Pro B cells in control mice were BrdU$^+$

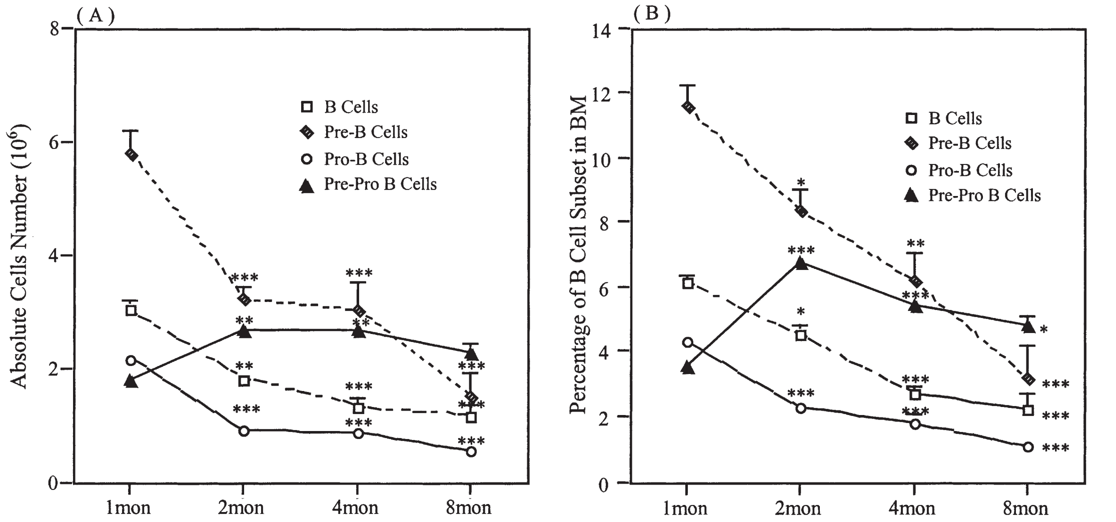

FIGURE 3 Age-related changes in the absolute number (A) and frequency (B) of B lineage subsets in NZB bone marrow. The average percentages among all nucleated cells and numbers per femurs and tibiae were determined by analyzing individual animals aged 1, 2, 4 and 8 months in three separate experiments. Statistical significance was determined by analysis of variance (ANOVA) with comparison to 1-month old BMC. *$p < 0.05$, **$p < 0.01$, ***$p < 0.001$.

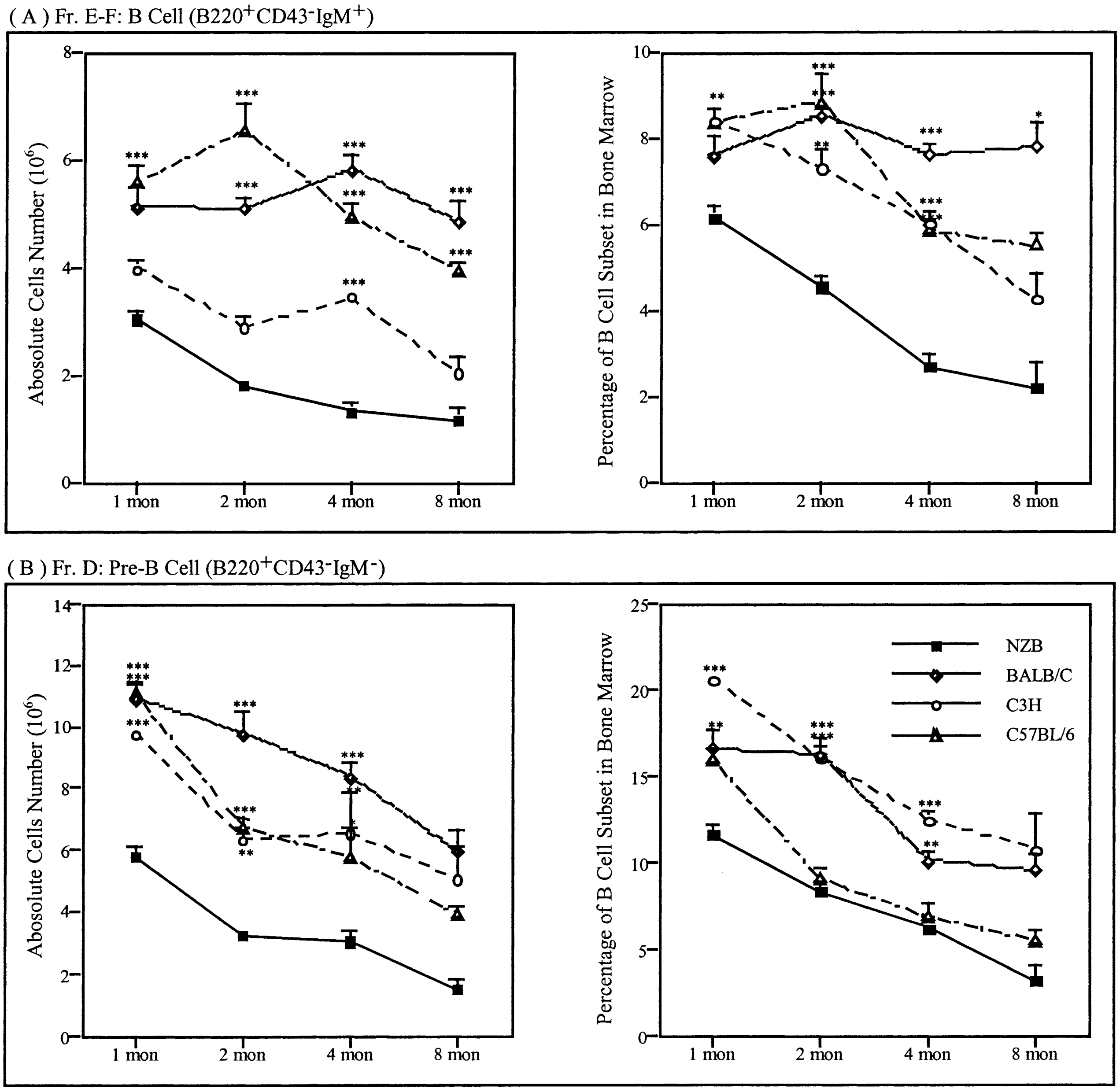

after 7 days of labeling, only 56% of the Pre–Pro B cells in NZB mice were BrdU$^{+}$ (Table I). These data suggest that increased proliferation does not account for the increased frequency and absolute numbers of Pre–Pro B cells in the bone marrow of NZB mice.

### The Rate of Pre–Pro B Cell Apoptosis in NZB Mice

The increased frequency of bone marrow Pre–Pro B cells in NZB mice could also be due to the increased survival of cells at this stage, resulting in an accumulation of cells in that compartment relative to normal mice. To address this issue, the frequency of cells undergoing spontaneous apoptosis was measured. The data in Fig. 7 show results from one of four representative experiments where the frequency of apoptotic Pre–Pro B cells was determined. Highly enriched populations of Pre–Pro B cells from adult NZB mice exhibited a much lower level of apoptosis (3.45 ± 0.23%) than similar preparations of cells from similarly aged BALB/c (9.1 ± 1.8%), and C57BL/6 mice (6.2 ± 1.3%) (Table II). Because apoptotic cells are rapidly removed from the bone marrow, the number of such cells detectable at any one time is low (Lu *et al.*, 1998). Therefore, a short-term culture system that allows for the accumulation and enumeration of apoptotic cells was used. IgM$^{+}$/HSA$^{+}$/CD19$^{+}$ depleted low-density BMC from similarly aged adult NZB and normal BALB/c mice were incubated for 24 h, and apoptosis was examined by Annexin V staining. Once again, the frequency of apoptotic Pre–Pro B cells in NZB mice was found to be markedly lower than in normal BALB/c mice (Fig. 7 and Table II). In addition, the finding that the

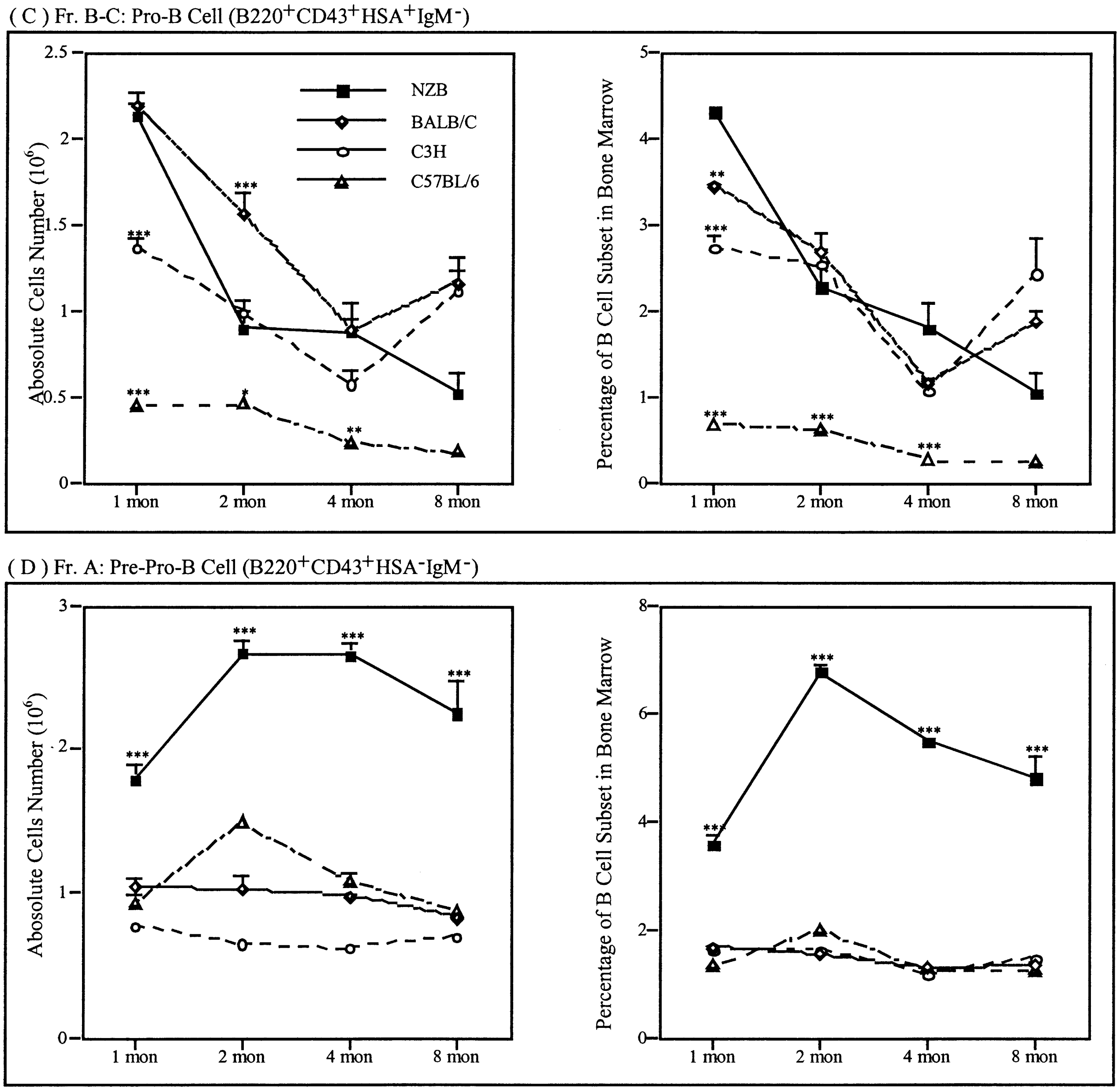

FIGURE 4 Comparison of proportion and numbers of (A): B cell (Fr. E–F), (B): Pre-B cell (Fr. D), (C): Pro-B cell (Fr. B–C), and (D): Pre–Pro B cell (Fr. A) between NZB mice and normal inbred strains. $*p < 0.05$, $**p < 0.01$, $***p < 0.001$ analysis of variance (ANOVA) with comparison to NZB mice.

expression of *Bcl-X_l* in Pre–Pro B cells in NZB mice is also higher than normal BALB/c mice (Fig. 8), supports the view that Pre–Pro B cells from NZB mice are relatively resistant to undergo apoptosis and, therefore, reflects a longer half-life of B cells at this stage of maturation.

### Gene Expression in Pre–Pro B Cells

To further clarify the mechanisms responsible for the abnormal accumulation of Pre–Pro B cells in NZB mice, we examined the expression of key B cell lineage genes involved in the process of B cell maturation and differentiation using a highly enriched population of Pre–Pro B cells from the bone marrow of 2-month-old NZB and age matched control normal BALB/c mice prepared as described. Figure 8 illustrates that the expression of the transcription factor BSAP in Pre–Pro B cells of NZB mice was down regulated, as were *Igα* (mb-1) and the *Igβ* (B29) genes. Levels of the expression of the surrogate light chain *λ5* and *Rag-2* were also lower in Pre–Pro B cells from NZB mice relative to levels seen in similar cells from control mice.

### DISCUSSION

NZB mice, as well as several other models of murine lupus models manifest abnormal patterns of B-lineage cell

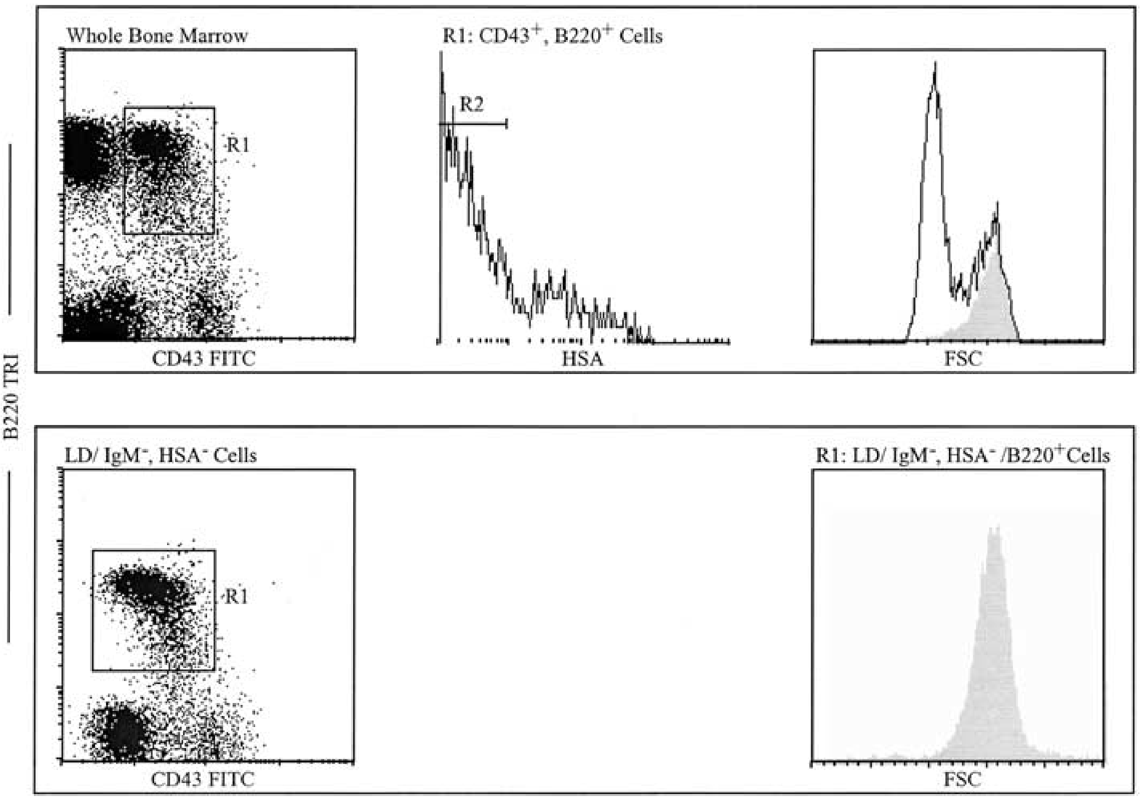

FIGURE 5 Whole BMC from NZB mice were enriched after discontinuous density gradent centrifugation; $IgM^+$ and $HSA^+$ cells were depleted by magnetic-beads as described in the "Materials and Methods" section. Note the high frequency of the Pre–Pro B cell subset (R1). A similar depletion was demonstrated with the control strains (data not shown).

development (Borchers *et al.*, 2000). Typically, NZB mice exhibit accelerated appearance and production of B lineage precursor cells during fetal and neonatal life (Jyonouchi *et al.*, 1983; Jyonouchi and Kincade, 1984). NZB mice develop large numbers of B cell precursors at an early stage of embryonic development, suggesting that hyperactive B cell formation continues for the first few weeks of life. By 5–6 months of age, however, the frequency and absolute numbers of Pre-B cells are markedly reduced when compared with age-matched normal murine strains (Jyonouchi *et al.*, 1982; Kruger and Riley, 1990).

Merchant *et al.* (1995; 1996) localized the decreases in the differentiation and maturation of B lineage cells to the Pre-B and immature B cell stages. However, it was not clear whether this decrease was manifest throughout the currently recognized stages of B cell maturation or initiated at the Pre-B or Pre–Pro B cell stage. Herein, we find that in fact there is an increase in the frequency of Pre–Pro B cells in the BM of NZB; this increase was most pronounced at 1 month of age and is sustained throughout the ages of the NZB mice studied, suggesting that there is a block in the maturation of B cells at the Pre–Pro B cell stage that leads to an accumulation of these cells with an associated decrease in the frequencies of the subsequent stages of B cell maturation. Rolink *et al.* have reported that $NK1.1^+$ cells may be included within

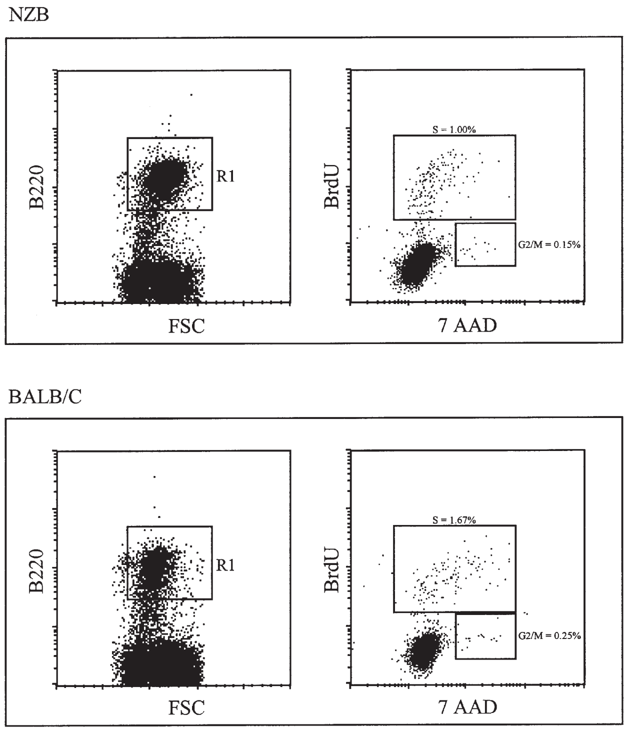

FIGURE 6 Cell cycle analysis of the Pre–Pro B cell frequency that have incorporated BrdU and total DNA. Data are representative of one of four experiments using a total of 2–4 mice per group.

TABLE I Expression of BrdU in Pre–Pro B Cells from NZB and Control Mice

| | $BrdU^+$ | |
|---|---|---|
| | 2 h | 7 Days |
| NZB | 0.89 ± 0.11 | 56.95 ± 0.95 |
| BALB/c | 1.94 ± 0.26* | 87.95 ± 0.37* |
| C57BL/6 | ND | 84.32 ± 0.56* |

The results from four separate experiments are presented. *$p < 0.001$ ANOVA test, ND: not done.

Pre–Pro B cells (Rolink *et al.*, 1996). As noted above, we found a similar frequency of $NK1.1^+$ cells in age-matched NZB and C57BL/6 mice. They are not expressed in BALB/c mice.

Numerous studies have described that allogeneic bone marrow transplantation (BMT) can be disease preventive and has been attempted for the treatment of both systemic and organ-specific autoimmune diseases in SLE models (Ikehara *et al.*, 1985; Ishida *et al.*, 1994; Adachi *et al.*, 1995; Mizutani *et al.*, 1995; Ikehara, 1998; Good, 2000; Good and Verjee, 2001). More recently, we have reported that stem cells from adult NZB mouse bone marrow exhibited defective T cell lineage development in fetal thymic organ culture (FTOC) (Hashimoto *et al.*, 2000). These findings suggest that autoimmune disease originates from intrinsic disorders of the HSCs themselves and in their developmental pathways, followed by autoreactive lymphocyte accumulation (Ikehara *et al.*, 1990; Ikehara, 2001). Our observation of an unusual expansion of Pre–Pro B cells in NZB mice is consistent with these studies.

It is believed that immune "tolerance" is accomplished, in part, through an educational phase of B lymphocyte development: autoreactive B cells are identified at an early maturational stage and effectively silenced. Importantly, depletion of immature B cells has been shown by studies that evaluated the numbers of cells that successfully traverse the immature to mature B cell stage of development daily (Lu and Osmond, 2000). The data indicate a high apoptotic index at the Pre–Pro B/Pro-B transition, many Pre–Pro B cells normally generate non-productive rearrangements and are diverted into a programmed cell death pathway. Our observation demonstrates that the level of spontaneous apoptosis of fresh and cultured Pre–Pro B cells in NZB mice are significantly lower than in normal mice, suggesting that some autoreactive B cells may escape from apoptosis to continue the maturation process. The general defect in apoptosis implicates the maturational arrest of the B cells in the pathogenesis of autoimmunity in NZB mice.

There are several mechanism(s) that either individually or in concert could account for the accumulation of the B lineage cells at the Pre–Pro B cell stage. These include prolonged persistence and half-life of cells at this stage of maturation, decreased sensitivity to undergo apoptosis (increased half-life) or dysregulation of genes that control

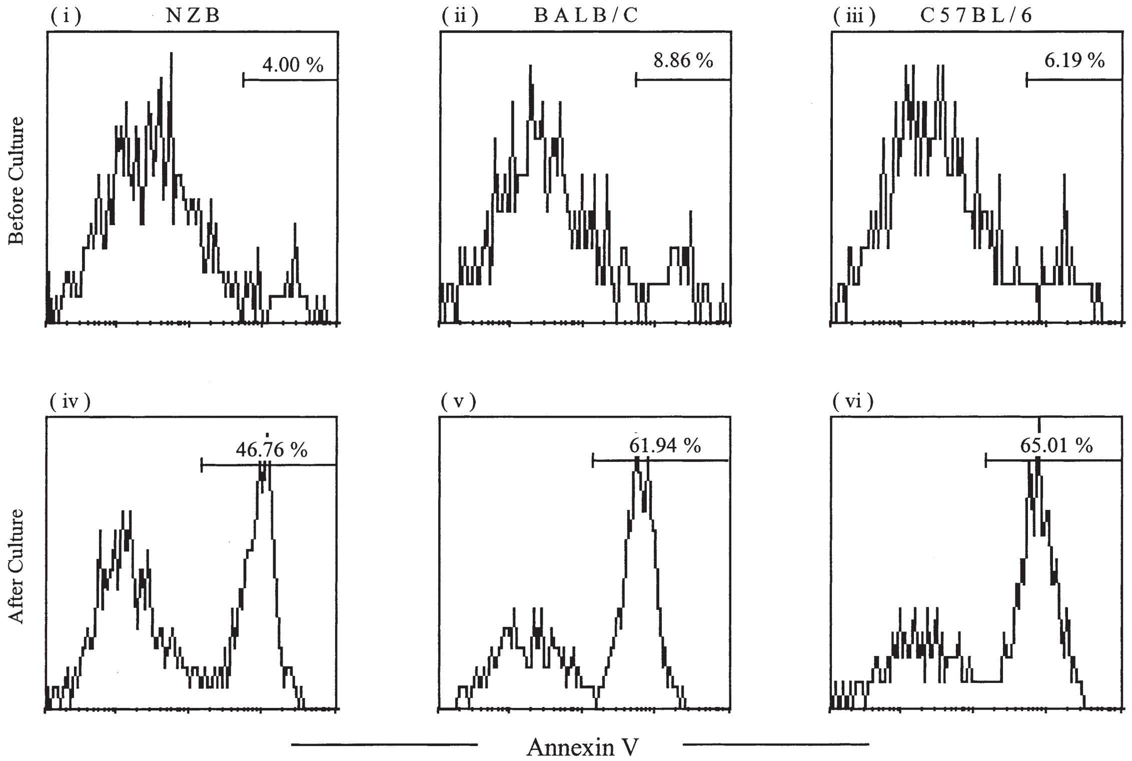

FIGURE 7 Frequency of Pre–Pro B cells from NZB, BALB/c mice and C57BL/6 mice undergoing spontaneous (i–iii) or following *in vitro* culture (iv–vi) apoptosis. Pre–Pro B cells were purified with magnetic-beads and flow cytometry profiles of Pre–Pro B cells (R1) and Annexin V expression histograms obtained of Pre–Pro B cells before or after 18 h following *in vitro* culture. Data are representative of one of three experiments using a total of 2–4 mice per group.

TABLE II Percentage of Annexin $V^+$ cells in Pre–Pro B cells

| | % of apoptosis in Pre–Pro B cells | |
|---|---|---|
| Strain | Before culture | After culture |
| NZB | 3.45 ± 0.23 | 49.46 ± 5.35 |
| BALB/c | 9.06 ± 1.77*** | 60.76 ± 8.34* |
| C57BL/6 | 6.21 ± 1.26** | 62.05 ± 8.48* |

*$p < 0.05$, **$p < 0.01$, ***$p < 0.001$ Unpaired ANOVA test. Data are representative of four independent experiments.

B cell differentiation and maturation. As we have demonstrated, the Pre–Pro B cells of NZB mice exhibit a much slower turnover rate and prolonged persistence, and resistance to apoptosis as compared to normal mice.

The dysregulation of genes controlling B cell differentiation/development is another facet of the abnormal Pre–Pro B cell population expansion. Essential to the understanding of the molecular basis of this pathology is the clear characterization of the rearrangement status of the immunoglobulin heavy chain (*IgH*) locus (Hardy and Hayakawa, 1991; 2001; Li *et al.*, 1993; 1996). The expression of $\mu$ is one of the earliest indication of B cell lineage commitment (Alessandrini and Desiderio, 1991; Schlissel *et al.*, 1991), reflecting the remodeling of chromatin structure to make the heavy chain locus accessible to rearrangements (Alt *et al.*, 1987). Transcripts of *Rag-1* and *Rag-2* gene are essential to *IgH* rearrangement; Pre–Pro B cells possess very low levels of mRNA from *Rag-1* and *Rag-2* genes and very little immunoglobulin D-J heavy chain rearrangement (Hardy and Hayakawa, 1991; Ehlich *et al.*, 1993; Li *et al.*, 1993). In this study, there was normal expression of the $\mu_0$ transcript in NZB mice, but *Rag-2* transcript was decreased in the Pre–Pro B fraction in NZB mice compared with normal mice, suggesting impairment of rearrangement in the Pre–Pro B fraction in the NZB mice.

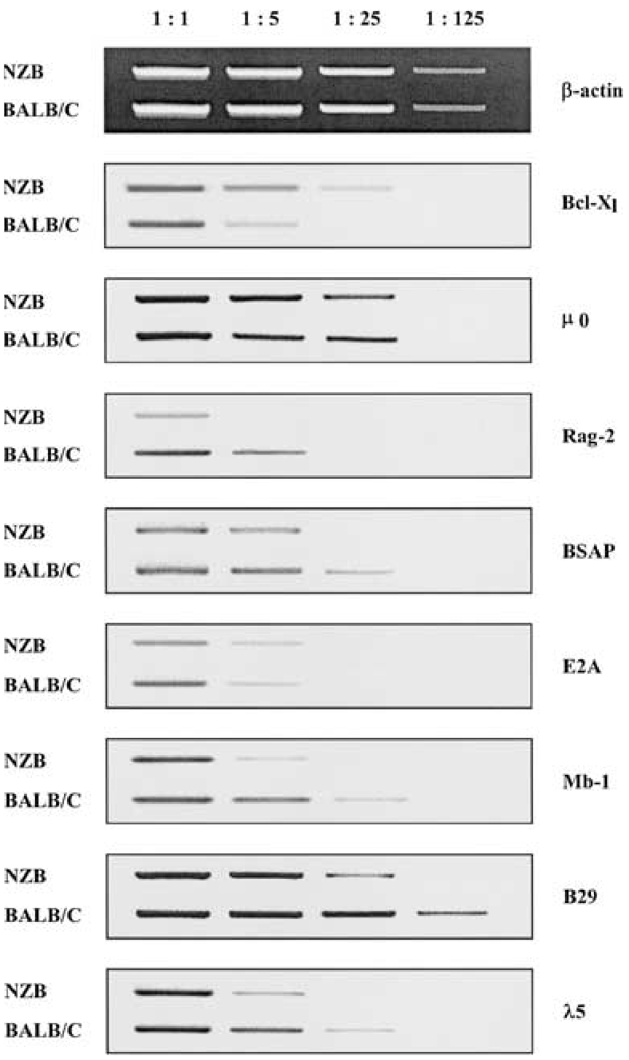

FIGURE 8 Expression of mRNA for *Bcl-X$_l$*, $\mu_0$, *Rag-2*, BSAP, *E2A*, *Ig$\alpha$*. *Ig$\beta$* and *$\lambda$5* in sorted $B220^+CD43^+CD19^-HSA^-$ (Pre–Pro B) cells from bone marrow of NZB and BALB/c mice. Total RNA isolation and RT-PCR assays were performed as described in "Material and Methods" section. Dilutions of cDNA were subjected to PCR amplification specific for *$\beta$-actin*, *Bcl-X$_l$*, $\mu_0$, *Rag-2*, BSAP, *E2A*, *Ig$\alpha$*. *Ig$\beta$* and *$\lambda$5* and the resulting products separated by electrophoresis on a 1.5% agarose gel containing ethidium bromide and visualized by UV light illumination.

The initiation of B-cell development critically depends on several transcription factors. B-cell-specific activator protein (BSAP, also termed B-lymphoid-specific transcription factor *pax5*) has been shown to play an essential role in early B cell development (Nutt *et al.*, 1999a,b). The absence of BSAP leads to an arrest in B cell development at the earliest stage before rearrangement of the *IgH* (Urbanek *et al.*, 1994) occurs. It has been shown that loss of BSAP affects the B lymphoid-restricted $V_H$-to-$D_HJ_H$ joining step of *IgH* assembly (Nutt *et al.*, 1997). BSAP appears to play a crucial role in B-lineage commitment (Nutt *et al.*, 1999a). In our experiments, impairment of BSAP was observed in NZB Pre–Pro B cells, supporting the thesis that dysregulation of the cascade of gene transcription is involved in the abnormality in NZB mice.

The initiation of B cell development also critically depends on the *E2A* gene which encodes two helix-loop-helix transcription factors, *E12* and *E47*. In the absence of these proteins, B cell development is arrested at the earliest stage, before $D_HJ_H$ rearrangement of the IgH chain occurs (Bain *et al.*, 1994; Zhuang *et al.*, 1994; Lin and Grosschedl, 1995). The level of *E2A* expression in Pre–Pro B cells were similar in NZB mice and BALB/c mice. *E2A* products are required for BSAP expression (Bain *et al.*, 1994), and therefore the impairment of BSAP in NZB mice is not likely due to altered levels of *E2A*. It has been suggested that the *E2A* gene products are involved in cell lineage commitment while BSAP is essential for progression of B cell development beyond the early Pro-B cell stage (Busslinger *et al.*, 2000). It has also been reported that a decrease in BSAP levels results in a loss of cell proliferation capability (Chong *et al.*, 2001), which is in accordance with our findings that BrdU incorporation is decreased in the NZB Pre–Pro B cell. Collectively, an abnormal decrease of BSAP expression may be responsible for the abnormal increase of the Pre-Pro B cells in NZB mice. We recognize, however, that this genetic analysis must be performed on isolated B cell subpopulations in a more quantitative fashion. Such work is in progress.

B cell antigen receptor complexes include membrane-bound immunoglobulin molecules non-covalently bound to the *Ig*$\alpha$ and *Ig*$\beta$ protein, respectively, the products of the *mb-1* and *B29* genes (Reth, 1992). In addition, *Ig*$\alpha$/*Ig*$\beta$ heterodimers are essential elements in Pre-B and B receptor signaling, and the role of *Ig*$\beta$ in B lymphopoesis before $\mu$ heavy chain synthesis has also been suggested using *Ig*$\beta$ knockout mice (Benlagha *et al.*, 1999). A recent report also suggests that signaling through *Ig*$\beta$ regulates locus accessibility for ordered Ig gene rearrangements (Maki *et al.*, 2000). In our study, *mb-1* and *B29* were also reduced in NZB mice Pre–Pro B cells. It is possible that the reduction of *mb-1* may be due to the reduced expression of BSAP because *mb-1* is positively regulated by BSAP (Busslinger *et al.*, 2000).

### Acknowledgements

Grant support provided by NIH CA 20816 and CA 20408.

### References

Adachi, Y., Inaba, M., Amoh, Y., Yoshifusa, H., Nakamura, Y., Suzuka, H., Akamatu, S., Nakai, S., Haruna, H., Adachi, M., Genba, H. and Ikehara, S. (1995) "Effect of bone marrow transplantation on antiphospholipid antibody syndrome in murine lupus mice", *Immunobiology* **192**, 218–230.

Akashi, K., Reya, T., Dalma-Weiszhausz, D. and Weissman, I.L. (2000) "Lymphoid precursors", *Curr. Opin. Immunol.* **12**, 144–150.

Alessandrini, A. and Desiderio, S.V. (1991) "Coordination of immunoglobulin DJH transcription and D-to-JH rearrangement by promoter-enhancer approximation", *Mol. Cell Biol.* **11**, 2096–2107.

Alt, F.W., Blackwell, T.K. and Yancopoulos, G.D. (1987) "Development of the primary antibody repertoire", *Science* **238**, 1079–1087.

Bain, G., Maandag, E.C., Izon, D.J., Amsen, D., Kruisbeek, A.M., Weintraub, B.C., Krop, I., Schlissel, M.S., Feeney, A.J., van Roon, M., van de Valk, M., te Riele, H.P.J., Berns, A. and Murre, C. (1994) "E2A proteins are required for proper B cell development and initiation of immunoglobulin gene rearrangements", *Cell* **79**, 885–892.

Benlagha, K., Guglielmi, P., Cooper, M.D. and Lassoued, K. (1999) "Modifications of Igalpha and Igbeta expression as a function of B lineage differentiation", *J. Biol. Chem.* **274**, 19389–19396.

Borchers, A., Ansari, A.A., Hsu, T., Kono, D.H. and Gershwin, M.E. (2000) "The pathogenesis of autoimmunity in New Zealand mice", *Semin. Arthritis Rheum.* **29**, 385–399.

Busslinger, M., Nutt, S.L. and Rolink, A.G. (2000) "Lineage commitment in lymphopoiesis", *Curr. Opin. Immunol.* **12**, 151–158.

Chong, S.Y., Zhang, M., Lin, Y.C., Coffman, F., Garcia, Z., Ponzio, N. and Raveche, E.S. (2001) "The growth-regulatory role of B-cell-specific activator protein in NZB malignant B-1 cells", *Cancer Immunol. Immunother.* **50**, 41–50.

Ehlich, A., Schaal, S., Gu, H., Kitamura, D., Muller, W. and Rajewsky, K. (1993) "Immunoglobulin heavy and light chain genes rearrange independently at early stages of B cell development", *Cell* **72**, 695–704.

Good, R.A. (2000) "Progress toward production of immunologic tolerance with no or minimal toxic immunosuppression for prevention of immunodeficiency and autoimmune diseases", *World J. Surg.* **24**, 797–810.

Good, R.A. and Verjee, T. (2001) "Historical and current perspectives on bone marrow transplantation for prevention and treatment of immunodeficiencies and autoimmunities", *Biol. Blood Marrow Transplant.* **7**, 123–135.

Hardy, R.R. and Hayakawa, K. (1991) "A developmental switch in B lymphopoiesis", *Proc. Natl Acad. Sci. USA* **88**, 11550–11554.

Hardy, R.R. and Hayakawa, K. (2001) "B cell development pathways", *Annu. Rev. Immunol.* **19**, 595–621.

Hashimoto, Y., Dorshkind, K., Montecino-Rodriguez, E., Taguchi, N., Shultz, L. and Gershwin, M.E. (2000) "NZB mice exhibit a primary T cell defect in fetal thymic organ culture", *J. Immunol.* **164**, 1569–1575.

Ikehara, S. (1998) "Autoimmune diseases as stem cell disorders: normal stem cell transplant for their treatment (Review)", *Int. J. Mol. Med.* **1**, 5–16.

Ikehara, S. (2001) "Treatment of autoimmune diseases hematopoietic stem cell transplantation", *Exp. Hematol.* **29**, 661–669.

Ikehara, S., Good, R.A., Nakamura, T., Sekita, K., Inoue, S., Oo, M.M., Muso, E., Ogawa, K. and Hamashima, Y. (1985) "Rationale for bone marrow transplantation in the treatment of autoimmune diseases", *Proc. Natl Acad. Sci. USA* **82**, 2483–2487.

Ikehara, S., Kawamura, M., Takao, F., Inaba, M., Yasumizu, R., Than, S., Hisha, H., Sugiura, K., Koide, Y., Yoshida, T.O., Ida, T., Imura, H. and Good, R.A. (1990) "Organ-specific and systemic autoimmune diseases originate from defects in hematopoietic stem cells", *Proc. Natl Acad. Sci. USA* **87**, 8341–8344.

Ishida, T., Inaba, M., Hisha, H., Sugiura, K., Adachi, Y., Nagata, N., Ogawa, R., Good, R.A. and Ikehara, S. (1994) "Requirement of donor-derived stromal cells in the bone marrow for successful allogeneic bone marrow transplantation. Complete prevention of recurrence of autoimmune diseases in MRL/MP-Ipr/Ipr mice by transplantation of bone marrow plus bones (stromal cells) from the same donor", *J. Immunol.* **152**, 3119–3127.

Jyonouchi, H. and Kincade, P.W. (1984) "Precocious and enhanced functional maturation of B lineage cells in New Zealand Black mice during embryonic development", *J. Exp. Med.* **159**, 1277–1282.

Jyonouchi, H., Kincade, P.W., Landreth, K.S., Lee, G., Good, R.A. and Gershwin, M.E. (1982) "Age-dependent deficiency of B lymphocyte lineage precursors in NZB mice", *J. Exp. Med.* **155**, 1665–1678.

Jyonouchi, H., Kincade, P.W., Good, R.A. and Gershwin, M.E. (1983) "B lymphocyte lineage cells in newborn and very young NZB mice: evidence for regulatory disorders affecting B cell formation", *J. Immunol.* **131**, 2219–2225.

Kruger, M.G. and Riley, R.L. (1990) "The age-dependent loss of bone marrow B cell precursors in autoimmune NZ mice results from decreased mitotic activity, but not from inherent stromal cell defects", *J. Immunol.* **144**, 103–110.

Li, Y.S., Hayakawa, K. and Hardy, R.R. (1993) "The regulated expression of B lineage associated genes during B cell differentiation in bone marrow and fetal liver", *J. Exp. Med.* **178**, 951–960.

Li, Y.S., Wasserman, R., Hayakawa, K. and Hardy, R.R. (1996) "Identification earliest B lineage stage in mouse bone marrow", *Immunity* **5**, 527–535.

Lian, Z., Toki, J., Yu, C., Hayashi, H., Yasumizu, R., Sugiura, K., Jin, T., Inaba, M., Hisha, H., Li, Y., Yu, W., Fan, H. and Ikehara, S. (1997) "Intrathymically injected hemopoietic stem cells can differentiate into all lineage cells in the thymus: differences between c-kit + cells and c-kit < low cells", *Stem Cells* **15**, 430–436.

Lian, Z., Feng, B., Sugiura, K., Inaba, M., Yu, C., Jin, T., Fan, T., Cui, Y., Yasumizu, R., Toki, J., Adachi, Y., Hisha, H. and Ikehara, S. (1999) "c-kit < low Pluripotent hemopoietic stem cells form CFU-S on day 16", *Stem Cells* **17**, 39–44.

Lin, H. and Grosschedl, R. (1995) "Failure of B-cell differentiation in mice lacking the transcription factor EBF", *Nature* **376**, 263–267.

Lu, L. and Osmond, D.G. (2000) "Apoptosis and its modulation during B lymphopoiesis in mouse bone marrow", *Immunol. Rev.* **175**, 158–174.

Lu, L., Smithson, G., Kincade, P.W. and Osmond, D.G. (1998) "Two models of murine B lymphopoiesis: a correlation", *Eur. J. Immunol.* **28**, 1755–1761.

Maki, K., Nagata, K., Kitamura, F., Takemori, T. and Karasuyama, H. (2000) "Immunoglobulin beta signaling regulates locus accessibility for ordered immunoglobulin gene rearrangements", *J. Exp. Med.* **191**, 1333–1340.

Merchant, M.S., Garvy, B.A. and Riley, R.L. (1995) "B220-bone marrow progenitor cells from New Zealand black autoimmune mice exhibit an age-associated decline in Pre-B and B-cell generation", *Blood* **85**, 1850–1857.

Merchant, M.S., Garvy, B.A. and Riley, R.L. (1996) "Autoantibodies inhibit interleukin-7-mediated proliferation and are associated with the age-dependent loss of pre-B cells in autoimmune New Zealand black mice", *Blood* **87**, 3289–3296.

Mizutani, H., Engelman, R.W., Kinjoh, K. and Good, R.A. (1995) "Gastrointestinal vasculitis in autoimmune-prone (NZW X BXSB)F1 mice: association with anticardiolipin autoantibodies", *Proc. Soc. Exp. Biol. Med.* **209**, 279–285.

Nutt, S.L., Urbanek, P., Rolink, A. and Busslinger, M. (1997) "Essential functions of Pax5 (BSAP) in pro-B cell development: difference between fetal and adult B lymphopoiesis and reduced V-to-DJ recombination at the IgH locus", *Genes Dev.* **11**, 476–491.

Nutt, S.L., Heavey, B., Rolink, A.G. and Busslinger, M. (1999a) "Commitment to the B-lymphoid lineage depends on the transcription factor Pax5", *Nature* **401**, 556–562.

Nutt, S.L., Vambrie, S., Steinlein, P., Kozmik, Z., Rolink, A., Weith, A. and Busslinger, M. (1999b) "Independent regulation of the two Pax5 alleles during B-cell development", *Nat. Genet.* **21**, 390–395.

Payne, K.J., Medina, K.L. and Kincade, P.W. (1999) "Loss of c-kit accompanies B-lineage commitment and acquisition of CD45R by most murine B-lymphocyte precursors", *Blood* **94**, 713–723.

Reth, M. (1992) "Antigen receptors on B lymphocytes", *Annu. Rev. Immunol.* **10**, 97–121.

Rolink, A., ten Boekel, E., Melchers, F., Fearon, D.T., Krop, I. and Andersson, J. (1996) "A subpopulation of B220+ cells in murine bone marrow does not express CD19 and contains natural killer cell progenitors", *J. Exp. Med.* **183**, 187–194.

Schlissel, M.S., Corcoran, L.M. and Baltimore, D. (1991) "Virus-transformed pre-B cells show ordered activation but not inactivation of immunoglobulin gene rearrangement and transcription", *J. Exp. Med.* **173**, 711–720.

Urbanek, P., Wang, Z.Q., Fetka, I., Wagner, E.F. and Busslinger, M. (1994) "Complete block of early B cell differentiation and altered patterning of the posterior midbrain in mice lacking Pax5/BSAP", *Cell* **79**, 901–912.

Zhuang, Y., Soriano, P. and Weintraub, H. (1994) "The helix-loop-helix gene E2A is required for B cell formation", *Cell* **79**, 875–884.

